# Comparative Analysis and Modeling of the Severity of Steatohepatitis in DDC-Treated Mouse Strains

**DOI:** 10.1371/journal.pone.0111006

**Published:** 2014-10-27

**Authors:** Vikash Pandey, Marc Sultan, Karl Kashofer, Meryem Ralser, Vyacheslav Amstislavskiy, Julia Starmann, Ingrid Osprian, Christina Grimm, Hendrik Hache, Marie-Laure Yaspo, Holger Sültmann, Michael Trauner, Helmut Denk, Kurt Zatloukal, Hans Lehrach, Christoph Wierling

**Affiliations:** 1 Max Planck Institute for Molecular Genetics, Department Vertebrate Genomics, Berlin, Germany; 2 Institute of Pathology, Medical University of Graz, Graz, Austria; 3 German Cancer Research Center, Heidelberg, Germany; 4 BIOCRATES Life Sciences AG, Innsbruck, Austria; 5 LKH Wagna, Department of Internal Medicine, Wagna, Austria; 6 Rheumatology and Clinical Immunology, Charité-University Medicine, Berlin, Germany; 7 Dahlem Centre for Genome Research and Medical Systems Biology, Berlin, Germany; 8 Hans Popper Laboratory of Molecular Hepatology, Division of Gastroenterology and Hepatology, Department of Medicine, Medical University of Vienna, Vienna, Austria; University College London, United Kingdom

## Abstract

**Background:**

Non-alcoholic fatty liver disease (NAFLD) has a broad spectrum of disease states ranging from mild steatosis characterized by an abnormal retention of lipids within liver cells to steatohepatitis (NASH) showing fat accumulation, inflammation, ballooning and degradation of hepatocytes, and fibrosis. Ultimately, steatohepatitis can result in liver cirrhosis and hepatocellular carcinoma.

**Methodology and Results:**

In this study we have analyzed three different mouse strains, A/J, C57BL/6J, and PWD/PhJ, that show different degrees of steatohepatitis when administered a 3,5-diethoxycarbonyl-1,4-dihydrocollidine (DDC) containing diet. RNA-Seq gene expression analysis, protein analysis and metabolic profiling were applied to identify differentially expressed genes/proteins and perturbed metabolite levels of mouse liver samples upon DDC-treatment. Pathway analysis revealed alteration of arachidonic acid (AA) and S-adenosylmethionine (SAMe) metabolism upon other pathways. To understand metabolic changes of arachidonic acid metabolism in the light of disease expression profiles a kinetic model of this pathway was developed and optimized according to metabolite levels. Subsequently, the model was used to study *in silico* effects of potential drug targets for steatohepatitis.

**Conclusions:**

We identified AA/eicosanoid metabolism as highly perturbed in DDC-induced mice using a combination of an experimental and *in silico* approach. Our analysis of the AA/eicosanoid metabolic pathway suggests that 5-hydroxyeicosatetraenoic acid (5-HETE), 15-hydroxyeicosatetraenoic acid (15-HETE) and prostaglandin D2 (PGD2) are perturbed in DDC mice. We further demonstrate that a dynamic model can be used for qualitative prediction of metabolic changes based on transcriptomics data in a disease-related context. Furthermore, SAMe metabolism was identified as being perturbed due to DDC treatment. Several genes as well as some metabolites of this module show differences between A/J and C57BL/6J on the one hand and PWD/PhJ on the other.

## Introduction

Nonalcoholic fatty liver disease (NAFLD) is a major cause of chronic liver damage in western countries and dependent on genetic and environmental factors. NAFLD can be considered as the hepatic manifestation of the metabolic syndrome and is linked to visceral obesity and has a higher prevalence among people with hyperlipidemia, hypertension, hyperglycemia and insulin resistance [1], [2]. Advanced stages of NAFLD, such as steatohepatitis, ultimately result in fibrosis and cirrhosis and can finally lead to liver failure or hepatocellular carcinoma (HCC) [3]. The importance of nonalcoholic steatohepatitis (NASH) in public health in the western world is demonstrated by the massive increase of obesity and type 2 diabetes mellitus, which are key components of the metabolic syndrome [4].

The molecular basis of complex diseases, such as NASH, is poorly understood and its analysis requires a detailed understanding of the underlying metabolic and regulatory processes on the molecular level. Steatohepatitis is characterized by alterations in the liver, such as steatosis, ballooning of hepatocytes, apoptosis, protein aggregates in hepatocytes (Mallory-Denk bodies), pericellular fibrosis, and predominantly polymorphonuclear granulocytic inflammation [3]. Certain features of steatohepatitis can be reproduced to a variable extent in different mouse models by various treatments like chronic intoxication with DDC (5-diethoxycarbonyl-1,4-dihydrocollidine), methionine- and choline-deficient diet, alcohol or high fat diet [5], [6].

Metabolites can be seen as end points of perturbations occurring at the gene level, so that changes of gene expression might to some extent also relate to changes in metabolite concentrations. The metabolic activity changes can be justified from transcript profiles based on the fact that mRNA is translated into a protein, e.g., working as an enzyme, thus changing metabolic flux of its catalyzed reaction [7]. Analysis of experimental data from cellular levels, i.e., transcriptomics, proteomics, fluxomics, and metabolomics show that there is not a high overall correlation between the abundance of RNA and its encoded protein, and between enzyme abundance and their respective catalyzed flux [7]. However, for regulated metabolic pathways the changes of RNA and protein abundances were in accordance with changes in reaction fluxes [7]. Hence, models integrating metabolic pathways and gene expression data may be used for *in silico* studies to predict changes in metabolite levels. Several tools exist to perform pathway analysis of expression and/or metabolic data that provide reasonable candidate pathways for subsequent modeling [8], [9]. Past studies already used combined analysis of gene expression and metabolite data for the identification of, e.g., a genetic network of liver metabolism [10], biomarkers of type 2 diabetes [11], and disease related active pathways [12].

Herein, we studied transcriptomics and metabolomics data of mice developing NASH-like phenotypic features. We fed three genetically different mouse strains A/J, C57BL/6J, and PWD/PhJ (henceforth AJ, B6 and PWD) a DDC-supplemented diet. These mouse strains belong to two different subspecies and therefore cover a broad genetic variety. AJ and B6 are classical laboratory mouse strains mainly of *Mus musculus domesticus* origin, whereas PWD is a wild-derived strain mainly of *Mus musculus musculus* origin. Liver samples of DDC-treated and untreated mouse strains were analyzed by RNA sequencing (RNA-Seq) providing comprehensive mRNA expression profiles. Furthermore, we quantified selected metabolites by mass spectrometry and some relevant proteins by reverse-phase protein array (RPPA). Pathway analysis of these data identified deregulated pathways such as, nucleotide, beta-alanine and histidine metabolism. Furthermore, strain-specific deregulation was found in the metabolism of S-adenosylmethionine (SAMe). Moreover, the metabolism of arachidonic acid was identified to be perturbed due to DDC-treatment irrespective of the strain. For further analysis and correlation of transcriptomics, protein and metabolite data, a kinetic model of the arachidonic acid metabolism was developed and fed with expression data to understand the metabolic changes. The arachidonic acid metabolic model was further used in an *in silico* study to obtain potential drug targets.

## Materials and Methods

### Mouse experiments and liver preparation

A/J, C57BL/6J and PWD/PhJ (abbreviated AJ, B6, and PWD) animals were obtained from Jackson Laboratories (The Jackson Laboratory, Maine, USA) and bred in the animal facility of the Medical University of Graz under specific pathogen free conditions. Eight weeks old male animals of each strain were fed either a standard (control) diet (Ssniff Spezialdiäten GmbH, Soest, Germany) or the standard diet supplemented with 0.1% DDC (5-diethoxycarbonyl-1,4-dihydrocollidine, Sigma-Aldrich, Vienna, Austria) for eight weeks under constant health monitoring.

All animal experiments were conducted according to the Austrian Animal Welfare Act. All experiments were approved by the Austrian ministry of science and research after review by the Austrian animal research committee under animal license number BMBWK-66.010/0047-BrGT/2005. Austrian law does not require individual institutional animal care and use committees for universities as all animal experimentation has to be pre-approved by the Austrian ministry of science and research.

Up to 4 animals were kept in individually ventilated cages at a monitored temperature of 20–24°C, with humidity between 40–70%, a constant 12 hour light and dark cycle and at least 50–60 air changes per hour. Animals were provided with water and rodent chow ad libitum. Animal health was monitored by daily visual cage inspection and weekly weight check of all experimental animals. Animals losing more than one third of body weight or showing other signs of distress due to DDC feeding were excluded from analysis and immediately sacrificed. All animals were sacrificed by cervical dislocation after anaesthesia by isoflurane inhalation. Liver tissues were harvested and samples of liver tissue were frozen in methyl-butane cooled by liquid nitrogen and subsequently stored in liquid nitrogen. RNA was prepared from frozen liver samples using the RNeasy Mini Kit (Qiagen GmbH, Hilden, Germany) according to manufacturers instructions.

### RNA-Seq experiments and data analysis

We performed transcriptome analysis of healthy and DDC-treated livers of three mouse strains AJ, B6, and PWD each, and three biological replicates per strain and condition. In total, 18 paired-end RNA-Seq libraries were prepared from 10 µg of total RNA using a strand-specific strategy and following the protocol described in [13]. The following ligation adapters and PCR primers were used: PE Adapter OligoMix (Cat.#1001782), PCR Primer PE 1.0 (Cat.#1001783), PCR Primer PE2.0 (Cat.#1001784), IndexPE Adapter Oligo Mix Cat.#1005711), PCR PrimerInPE 1.0, (Cat.#100571), PCR PrimerInPE 2.0, (Cat.#1005713). Sequencing was carried out on the GAIIx platform (Illumina) by running 2×51 cycles according to the manufacturer instructions. Sequencing reads were aligned to the mm9 assembly of the mouse reference genome using BWA [14]. Gene levels were then quantified in reads per kilobase of exon model per million mapped reads [15] and using the Ensembl v.53 (*Mus musculus*) annotation. Identification of differentially expressed genes (DEGs) was performed with the R-package “edgeR” [16]. For identification of DEGs three biological replicates of each mouse strain AJ, B6, and PWD for control and DDC-treated states were used. DEGs were identified in two ways: (i) “strain-wise” and (ii) “irrespective of strains”. For strain-wise identification DEGs between DDC-treated and control states were computed separately for each mouse strain AJ, B6, and PWD, while for the identification irrespective of strains control and DDC-treated states were analyzed across all three mouse strains together. Using edgeR p-values were calculated and adjusted for multiple testing using the Benjamini-Hochberg procedure implemented in edgeR. Principal components analysis (PCA) was performed to investigate any grouping of strain-wise DEGs.

### Metabolomics analysis

#### Acylcarnitines, Sphingomyelins, Hexoses, Glycerophospholipids (FIA-MS/MS)

To determine the concentration of acylcarnitines, sphingomyelins, hexoses and glycerophospholipids in liver homogenate the AbsoluteIDQ kit p150 (Biocrates Life Sciences AG) was prepared as described in the manufacturer's protocol. In brief, 10 µl of liver homogenate was added to the centre of the filter on the upper 96-well kit plate, and the samples were dried using a nitrogen evaporator (VLM Laboratories). Subsequently, 20 µl of a 5% solution of phenyl-isothiocyanate was added for derivatization. After incubation, the filter spots were dried again using an evaporator. The metabolites were extracted using 300 µl of a 5 mM ammonium acetate solution in methanol. The extracts were obtained by centrifugation into the lower 96-deep well plate followed by a dilution step with 600 µl of kit MS running solvent. Mass spectrometric analysis was performed on an API4000 QTrap tandem mass spectrometry instrument (Applied Biosystems/MDS Analytical Technologies) equipped with an electro-sprayionization (ESI)-source using the analysis acquisition method as provided in the AbsoluteIDQ kit. The standard FIA-MS/MS method was applied for all measurements with two subsequent 20 µl injections (one for positive and one for negative mode analysis). Multiple reaction monitoring (MRM) detection was used for quantification applying the spectra parsing algorithm integrated into the MetIQ software (Biocrates Life Sciences AG).

#### Prostanoids, oxidized fatty acids (LC-MS/MS)

Prostanoids – a term summarizing prostaglandins (PG), thromboxanes (TX) and prostacylines – and oxidised fatty acid metabolites were analyzed by LC-ESI-MS/MS [17] by online solid phase extraction (SPE)-LC-MS/MS with an API4000 QTrap tandem mass spectrometry instrument (Applied Biosystems/MDS Analytical Technologies) in negative MRM detection mode. In brief, filter spots in a 96 well plate were spiked with internal standard; 20 µl of sample were added and extracted with aqueous methanol, the individual extracts then were analysed. Data of prostanoids and oxidized fatty acids were quantified with Analyst 1.4.2 software (Applied Biosystems) and finally exported for statistical analysis.

### Protein preparation

Proteins were isolated from fresh-frozen tissue using T-Per extraction reagent (Pierce Biotechnology, Inc., Rockford, IL, USA) according to the manufacturer's recommendations, with the addition of the following inhibitors: complete mini protease inhibitor cocktail, staurosporin and PhosStop (Roche, Mannheim, Germany). For protein extraction from tissue (30–40 mg) the Qiagen Tissuelyser (Qiagen, Hilden, Germany) was used. Samples were isolated in 10 µl protein lysis buffer per 10 mg sample. Protein concentrations were determined using the bicinchoninic acid assay reagents (Pierce Biotechnology, Inc.). Protein lysates were stored at −80°C.

### Reverse-phase protein array (RPPA) processing and data analysis

Protein lysates were diluted using protein lysis puffer (2 µg/µl). After adding Tween 20 (0.05%, v/v) protein lysates were printed onto nitro-cellulose coated glass slides (Oncyte Nitrocellulose Film Slides, Grace Bio-Labs, Blend, OR, USA) using the Aushon 2470 solid-pin tool arrayer (Aushon Biosystems, Billerica, MA, USA). Antibody incubation and antibody-mediated signal amplification were performed as described [18]. Slides were scanned with the Odyssey NIR scanner (LI-COR Biosciences, Bad Homburg, Germany). Image analysis was carried out with GenePix-Pro 6.0 (Axon Instruments, Sunnyvale, USA). Data sets were analyzed using the RPPAnalyzer package [19]. Quantification results were normalized to the Fast Green FCF staining of total proteins as well as to the median antibody binding signal levels [20].

### Antibody validation for RPPA

To verify antibody specificities, a pool of protein lysates from cancer and benign tissues was analyzed by SDS-PAGE followed by Western Blotting. 25 µg of the protein lysate was used and a standard near-infrared detection was applied as described in [18]. Antibodies with a predominant single band in the expected size range were selected for further RPPA analysis. Antibodies were purchased from Cell Signaling Technology (Danvers, MA, USA).

### Model Initialization

An example of mapping RNA-Seq or RPPA data to enzyme concentration is described below. Assume a mass action kinetic equation,
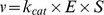
where *v* is the flux, *k_cat_* is the enzymatic turnover number, and *E* and *S* are the enzyme and the substrate concentrations, respectively. The aforementioned equation is transformed in equation, 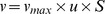
where *v_m_*_ax_ is the maximum rate and *u* is a unitless quantity representing a ratio. For the initialization of the scaling factor *u* with RNA-Seq or RPPA data we use 1 for the control state and the ratio between treatment and control for the DDC-supplemented state. The ratio is computed based on the experimental transcriptomics and proteomics data form mice fed 8 weeks with a DDC-supplemented diet and their respective controls. The state after 8 weeks is assumed as a quasi steady state also regarding the quantified metabolite concentrations.

### Modelling and kinetic parameter optimization

The mathematical model of the arachidonic acid (AA)/eicosanoid pathway was developed in PyBioS [21], [22] based on information from KEGG and a survey of literature [23]–[26]. Parameter optimization of the mathematical model was performed with COPASI [27]. COPASI provides a number of different algorithms for optimization of a predefined objective function. We used the genetic algorithm in our analysis to minimize the objective function
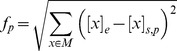
where *x* is a given metabolite of a set of metabolites *M* of our metabolic model, [*x*]_e_ and [*x*]_s_ are concentration values of a metabolite *x* from experiments and simulations, respectively, and **p** is a set of kinetic parameters. 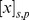 is the steady state concentration of metabolite *x* during simulation which depends on **p**. 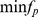 is an optimization problem which minimize *f* over **p**.

## Results

Liver samples of DDC-treated and untreated mouse strains (AJ, B6 and PWD) were phenotypically characterized and analyzed by RNA-Seq providing comprehensive mRNA expression profiles, by mass spectrometry of selected metabolites, and by RPPA analysis of relevant proteins.

### Phenotypic characterization

**Figure 1:**
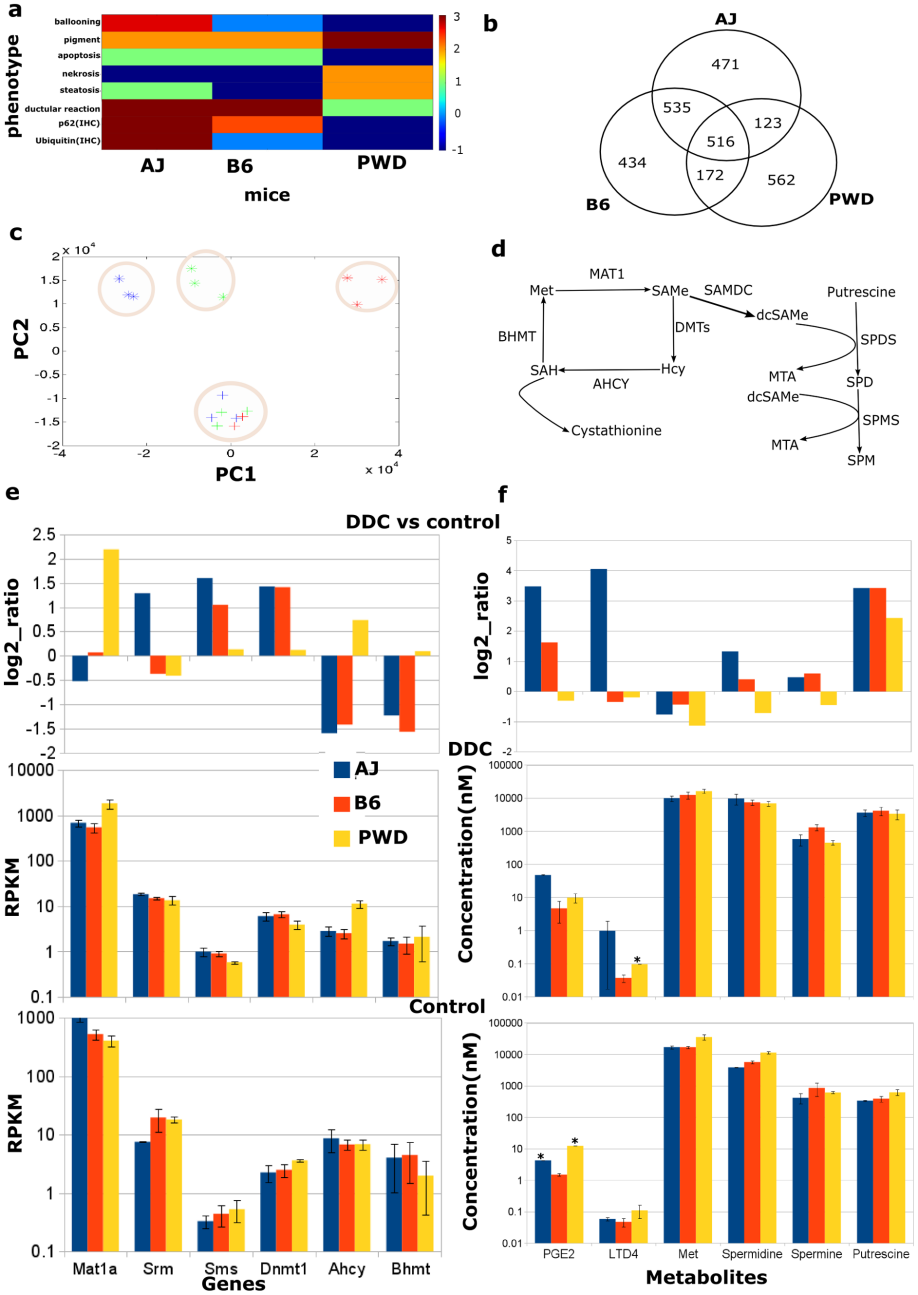
Analysis of phenotypic and omics data. **a**) Qualitative scoring of histological phenotypes of the mouse liver samples. Score -1, absent; score 0, minimal; score 1, mild; score 2, moderate; score 3, severe changes compared to healthy liver tissue. Immunohistochemistry, IHC. **b**) Venn diagram of differentially expressed genes due to DDC treatment in AJ, B6, and PWD mice. **c**) Principle component analysis (PCA) of 2813 genes that were found differentially expressed for at least one mouse strain due to DDC-treatment. * and + indicate control and DDC mice, respectively; red, green and blue represent AJ, B6, and PWD mice, respectively. Principle component 1 (PC1) explains 43% and PC2 29% of the data. **d**) S-adenosylmethionine (SAMe) metabolism. Methionine (Met) is converted to SAMe by the enzyme methionine adenosyltransferase (MAT1). SAMe is converted into S-adenosylhomocysteine (SAH) by DNA-methyltransferase (DMTs) and SAH hydrolase (AHCY) with homocysteine (Hcy) as an intermediate. SAH is substrate for Met formation by betaine-homocysteine methyltransferase (BHMT). SAMe can also be converted into spermine (SPM) via decarboxylated SAMe (dcSAMe) and spermidine (SPD) catalyzed by SAMe decarboxylase (SAMDC), SPD synthase (SPDS), and SPM synthase (SPMS). This pathways is regulated by putrescine, which activates SAMDC. **e**) Arithmetic mean values of RPKM values of aforementioned genes for liver samples of control and DDC-treated mice. Error-bars indicate standard deviations. The bar chart shows log2-ratios of RPKM values of DDC-treated vs control. The genes *Mat1a*, *Srm*, *Sms*, *Dnmt1*, *Ahcy*, and *Bhmt* encode the enzymes MAT, SPDS, SPMS, DMTs, AHCY and BHMT, respectively. **f**) Bar chart of median concentrations of the metabolites prostaglandin D2 (PGD2), leukotriene D4 (LTD4), methionine (Met), spermidine, spermine, and putrescine. Error-bars indicate median absolute deviations. * indicates samples without a replicate.

Phenotypes of liver samples of all three mouse strains after eight weeks of a DDC-supplemented diet were characterized by histological examination and formation of Mallory-Denk bodies using immunohistochemical analysis of ubiquitin and p62 (Fig. 1a). Steatosis was observed to a medium degree in AJ and PWD while it was absent in B6. Ballooning of the hepatocytes and the occurrence of ubiquitin and p62 containing protein aggregates was most pronounced in AJ. Based on the degree of steatohepatic features AJ and B6 were categorized as high and low susceptible, respectively, while PWD is resistant.

### Identification of differentially expressed genes using RNA-Seq data

Using RNA-Seq data differentially expressed genes (DEGs) in response to DDC treatment were computed (i) “strain-wise” and (ii) “irrespective of strains” as described in the material and methods section. The total number of DEGs from strain-specific analysis was very similar between the strains with 1,645, 1,657 and 1,373 for AJ, B6 and PWD, respectively (Fig. 1b). In total, 516 genes were deregulated in all three strains in response to DDC feeding, whereas 471 DEGs were found exclusively in AJ (Fig. 1b, Table S1). We call these 471 genes susceptibility genes since this strain shows the most pronounced steatohepatitis phenotype. In addition, to address the DDC treatment effect a principle component analysis (PCA) was performed based on 2,813 genes (Table S2), which were differentially expressed in at least one mouse strain due to DDC treatment (Fig. 1c). While healthy mice show strain-specific expression profiles in PCA, all mouse profiles from DDC treatment group together, implying a similar overall response due to DDC treatment irrespective of the strain (Fig. 1c). Besides the strain-specific analysis, DEGs were also computed irrespective of strains. This analysis yields in total 4,215 DEGs in response to DDC treatment (Table S3).

### Analysis of proteomics data

For a screening of changes of the proteome some specific proteins were also analyzed by reverse phase protein arrays (RPPA; Table S4). Notably, the protein expression levels of the liver-type fatty acid-binding protein (Fabp1) are decreased in all strains after DDC-feeding. This observation is in agreement with data from human samples showing an underexpression in mild and progressive stages of NASH but paradoxically an overexpression in simple steatosis [28]. Furthermore, keratin 8 (Krt8) and keratin 18 (Krt18) were found to be upregulated after DDC treatment. Both proteins are major components of the hepatocyte cytoskeleton. Other proteins that were found to be upregulated are glutathione S-transferase alpha (Gsta1) and glutathione S-transferase mu (Gstm1) that are both involved in detoxification processes and catalyze the conjugation of reduced glutathione to xenobiotics. Notably, the level of glutathione S-transferase alpha upregulation in AJ and B6 mice is much higher compared to PWD. Moreover, the protein expression level of the cytochrome Cyp2e1 level is found to be down-regulated in PWD, while it is not changed in B6 and AJ mice. In addition to general protein amounts, also phosphorylated states of the key signaling proteins Erk1 (Mapk3), Akt1 and Stat3 were measured reflecting the activity states of their respective signaling pathways. All of them were found to be induced due to DDC treatment.

### Pathway analysis of metabolic profiles and gene expression data

Besides gene expression and proteomics data, also 44 metabolites were identified and quantified by mass spectrometry. Differences in metabolite concentrations due to DDC treatment were judged by t-test (Table S5). A pathway analysis of metabolic profiles and gene expression data was performed with ConsensusPathDB to identify steatohepatitis-specific pathways [29]. Pathway over-representation analysis of the aforementioned 471 susceptibility genes obtained only in AJ identified nucleotide, histidine, beta-alanine, purine metabolism, apoptosis and steroid hormone biosynthesis upon the top-ranked deregulated pathways (Table S6). Within the beta-alanine pathway we found *Srm* encoding spermidine synthase (SPDS) as being upregulated in AJ (2.44-fold in AJ compared to 0.77-fold and 0.75-fold in B6 and PWD, respectively, Tab. S2) as well as the related metabolites spermidine (SPD) and spermine (SPM) that form a module of the hepatic S-adenosylmethionine (SAMe) metabolism (Fig. 1d, [30]). SAMe is needed for methylation of DNA, RNA and lipids, and synthesis and catabolism of SAMe is tightly regulated and changes in SAMe level might lead to fatty liver disease and the development of HCC [30].

Gene expression and metabolite concentrations of hepatic SAMe metabolism were found to be affected in the DDC-treated state (Fig. 1e and 1f). Expression of the genes methionine adenosyltransferase 1 alpha (*Mat1a*), spermine synthase (*Sms*), DNA methyltransferase 1 (*Dnmt1*), adenosylhomocysteinase (*Ahcy*), betaine-homocysteine methyltransferase (*Bhmt*), and spermidine synthase (*Srm*) and concentration changes of the related metabolites spermidine, spermine and putrescine show a different behavior between AJ, B6 and PWD. Gene expression and metabolic profiles of hepatic SAMe metabolism imply that PWD has an opposite response compared to AJ and B6 after feeding a DDC-supplemented diet which resembles the differences in steatohepatitis phenotypes of the strains. The enzyme SAMe decarboxylase (SAMDC) is activated by putrescine and upregulation of spermidine synthase (SPDS) and spermine synthase (SPMS) might explain the increased concentration of spermidine (SPD) and spermine (SPM) in AJ. A higher concentration of putrescine in AJ and B6 lowers the *K_m_* of SAMDC activating polyamine synthesis [30] that may affect the concentration of SAMe. This might affect methylation of various substrates such as DNA, RNA and lipids that might be one reason in NASH disease development. However, it remains difficult to judge the difference in degree of steatoheapatitis since the expression data and metabolic profiles of AJ and B6 show a similar response to the DDC-supplemented diet.

NAFLD has been defined as a metabolic disease associated with the insulin-resistance syndrome [1]. A genome-scale metabolic network of the mouse comprising 3,724 reactions, 2,774 metabolites, and 1,415 enzyme coding genes was used as a reference to identify metabolic pathways of related genes [31]. Irrespective of strain-specific effects, a group of 288 genes (out of 4,215 DEGs) coding for metabolic enzymes of the genome-scale metabolic network were identified as differentially expressed (edgeR p-value <1e-6 over all three strains) after DDC treatment (Table S7). This set of 288 genes was subsequently used in a pathway analysis with ConsensusPathDB to identify affected metabolic pathways due to DDC treatment (Table S8). Furthermore, a set of 19 differentially regulated metabolites (t-test p-value <0.05, Table S5) was used for a metabolite-based over-representation analysis with ConsensusPathDB (Table S9). Both, the pathway analysis of the transcriptomics as well as the metabolic data set, yield the arachidonic acid pathway upon others as being deregulated after DDC treatment (p-value 2.6e-4 and 1.3e-4, respectively, Table S8 and S9). Analysis of metabolites of the arachidonic acid/eicosanoid pathway yield also significant changes due to DDC treatment of some metabolites of this pathway. The abundances of four metabolites of this pathway are significantly altered due to DDC-treatment: PGD2, 5-HPETE, 15-HETE, and 15-HPETE (p-value<0.05, Table S4). An increased activity of the arachidonic acid pathway could also be observed in the metabolic data. Based on these observations we selected the arachidonic acid metabolism as a candidate for further *in silico* analysis.

### Model of the arachidonic acid/eicosanoid metabolic pathway

Arachidonic acid (AA) is a fatty acid usually coming from dietary animal sources or being synthesized from dietary linoleic acid. AA is present in cell membranes as a part of phospholipids. AA is released from phospholipids by phospholipase A2 (PLA2) and subsequently it acts as a precursor of prostaglandins and their related compounds, the prostacyclins, thromboxanes and leukotrienes. Several physiological effects of prostaglandins are described in the literature, such as inflammatory response, pain, fever, blood pressure, blood clotting, and regulation of sleep/wake cycle [32], [33]. Cyclooxygenase-1, also known as prostaglandin H2 synthase 1 (Ptgs1), catalyzes the reaction of arachidonate to prostaglandin H2 (PGH2), which is the precursor of other prostaglandins, prostacyclins, and thromboxanes. The enzyme 5-lipoxygenase (Alox5) produces 5-HPETE, which is a precursor of leukotrienes. The metabolites PGD2, 5-HPETE, 15-HETE and 15-HPETE have been observed to be significantly (p<0.05) changed in concentration due to DDC treatment in the liver samples of all mouse strains (Table S5).

**Figure 2:**
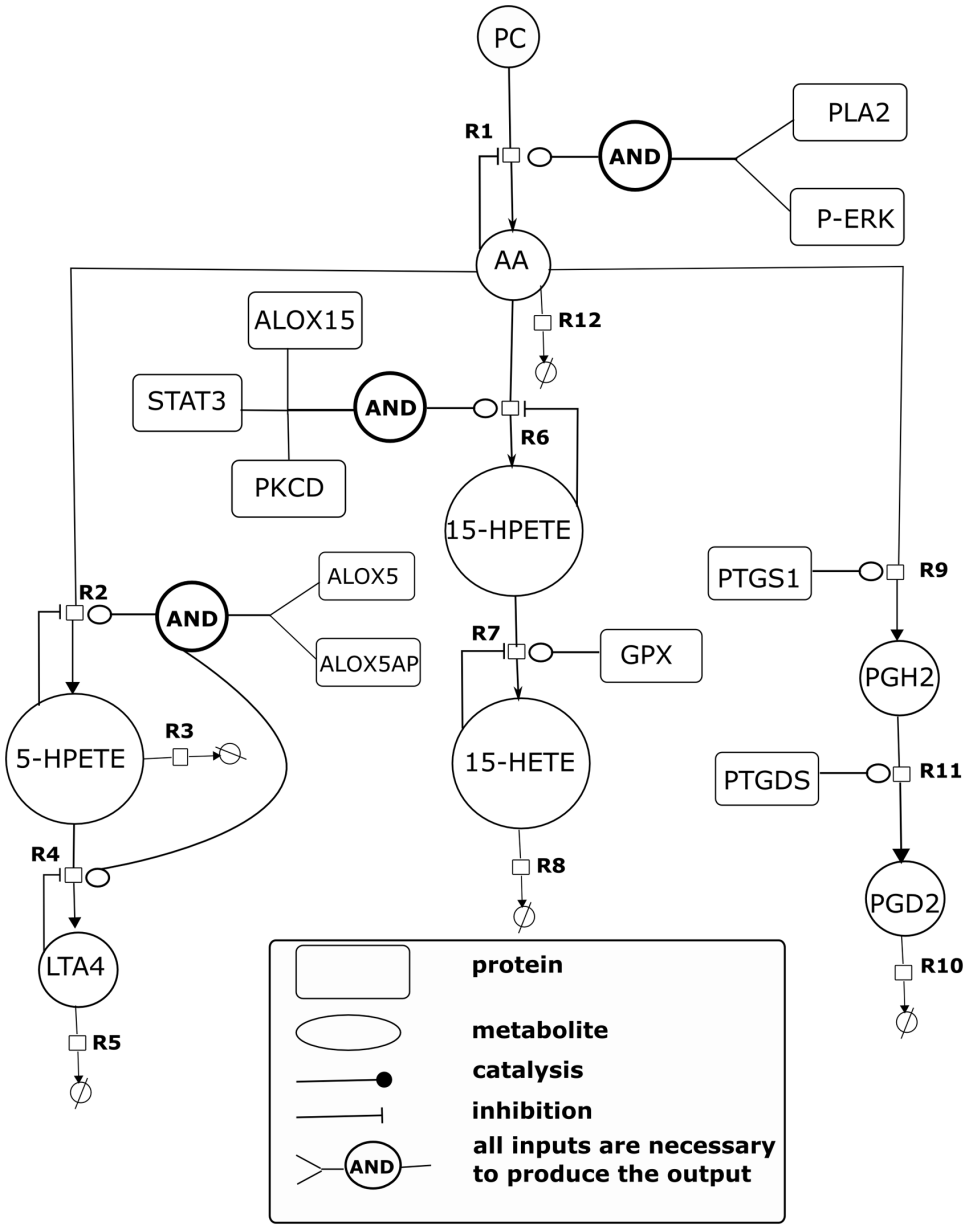
Model of arachidonic acid/eicosanoid metabolism. The model has three branches. Through the first branch arachidonic acid (AA) forms leukotriene A4. In the second branch 15-HPETE and 15-HETE are derived from AA, while in the third branch AA forms prostaglandin D2. See the results section for a detailed description of the model inhibitory links. Phosphatidylcholine, PC; arachidonic acid, AA; phospholipase A2, PLA2; phosphorylated ERK, P-ERK; cyclooxygenase-1, also known as prostaglandin G/H synthase 1, PTGS1; protein kinase C delta, PKCD; signal transducer and activator of transcription 3, STAT3; arachidonate 15-lipoxygenase, ALOX15; arachidonate 5-lipoxygenase, ALOX5; arachidonate 5-lipoxygenase-activating protein, ALOX5AP; prostaglandin H2, PGH2; prostaglandin D2, PGD2; prostaglandin D2 synthase, PTGDS; 15-hydroxyeicosatetraenoic acid, 15-HETE; 5- and 15-hydroperoxyeicosatetraenoic acid, 5- and 15-HPETE; leukotriene A4, LTA4; glutathione peroxidase, GPX.

There are only a few kinetic models of the AA metabolism available in the literature to study anti-inflammatory drugs of human polymorphonuclear leukocytes [23] and macrophage cells [24]. Here, an *in silico* model of the AA metabolism in mouse liver was developed based on information from KEGG [26] and a biochemical text book [25] to study the regulation of the perturbed metabolites using the related transcriptome and protein data. The structure of the model is depicted in Fig. 2.

The downstream synthesis of prostaglandins and leukotrienes is initiated by signaling and subsequent release of AA catalyzed by phospholipase A2 (PLA2). In endothelial cell-pericyte co-cultures of rat cells it has been shown that PLA2 is induced through the activation of the MAPK/ERK cascade [34]. PLA2 is involved in inflammation driven liver fibrosis as a key feature of progressive NASH and macrophage PLA2 deficiency prevented activation of hepatic stellate cells and infiltration of F4/80-positive macrophages [35]. 15-lipoxygenase (ALOX15) is an important regulator of inflammation and apoptosis and its expression is regulated by a cytosolic signaling complex with protein kinase C delta (PKCD) and phosphorylated STAT3 [36]. Thus, to describe the activity of PLA2 and PKCD, respectively, RPPA data of phosphorylated ERK and STAT3 was used subsequently for the modeling (Table S4).

The eicosanoid pathway is regulated by several feedback mechanisms (see Fig. 2). The release of AA is controlled by an inhibitory link between AA and phospholipase A2 (PLA2, R1 in Fig. 2, [37]). PLA2 is activated by phosphorylated ERK (p-ERK). Presence of both PLA2 and p-ERK is necessary to drive the metabolic conversion of phosphatidylcholine (PC) into AA (see R1 in Fig.2). To reflect the inhibition of PLA2 by AA an inhibitory feedback link is added to R1. The activity of 5-lipoxygenase (ALOX5) catalyzing the production of 5-HPETE and leukotriene A4 (LTA4) is controlled by product inhibition and by the ALOX5-activating protein ALOX5AP (R2, R4, [38], [39]). To convert AA into 15-HPETE the presence of both ALOX15 and a complex with PKCD and STAT3 are necessary. The production of 15-HPETE is also controlled by an inhibitory link between 15-HPETE and ALOX15 which is added to R6 (Fig. 2, [23]). Synthesis of 15-HPETE and PGH2 are controlled by feedback inhibition and glutathione peroxide and PTGS1, respectively (R7 and R9, [23], [40].

We hypothesize that changes of metabolite concentrations of the AA pathway can be explained by changes in mRNA expression. To address this hypothesis, changes in *V_max_* values of enzymatic reactions were approximated by fold changes of RNA-Seq expression of the respective enzymes due to DDC treatment. The fold change of PLA2 was approximated by *Pla2g4a* which was expressed at a low level (<1 RPKM, [41]) but not differentially (Table S10). Therefore, we hypothesize that PLA2 is at least present to trigger AA release upon its activity state, but it is not differentially expressed due to DDC treatment. Similarly, ALOX5 and ALOX15 were approximated by *Alox5* (p-value = 0.13) and *Alox15* (p-value = 0.62), respectively, which were expressed at low level (<1 RPKM) and we hypothesize that the expression of these genes were not affected due to DDC-treatment. PTGDS is approximated by *Ptgds* expression, but it was not significantly changed due to DDC treatment (p-value = 0.64). If there are several isoenzymes that can catalyse the same reaction the most significantly differentially expressed gene was chosen. For instance, GPX can be approximated by *Gpx1*, *Gpx2*, *Gpx3*, *Gpx6* and *Gpx7*. Since *Gpx3* was significantly affected (p = 2.66e-41) and highly expressed it was used to initialize the GPX expression in the model. Fold changes of the enzymes PTGS1, ALOX5AP and PKCD were approximated by expression values of *Ptgs1*, *Alox5ap* and *Prkcd*, respectively. Within our experiments the protein amount of phosphorylated ERK (p-ERK) and phosphorylated STAT3 (p-STAT3) were measured for both control and DDC-treated mice using the reverse phase protein array (RPPA) technology. For this we made use of respective proteomics data and initialized the model components p-ERK and p-STAT3 based on RPPA data. Since p-ERK and p-STAT3 were upregulated due to DDC treatment (Table S4; fold changes of p-ERK and p-STAT3 in AJ, B6, and PWD are 1.90, 2.09, 1.98, and 1.41, 1.42, 2.03, respectively) the effect of p-ERK on PLA2 is modeled by the RNA-Seq expression value of *Pla2g4a* for the DDC-treatment simulation and the p-ERK ratio. Similarly, the effect of the complex formation of PKCD and p-STAT3 on the ALOX15 activity was described by the RNA-Seq expression value of ALOX15 of the DDC-treatment state and the fold change of p-STAT3 and PKCD, respectively. Phosphatidylcholine (PC) was modeled as a fixed component and as its initial concentration the measured concentration of phosphatidylcholine C33:2 was used (Table 1). The experimentally identified fold changes of all enzymes are summarized in Table 1 and were used to approximate the kinetic parameters of the respective reactions. The kinetic parameters of the model were optimized by an objective function integrating the experimental data (see parameter optimization in section Material and Methods). Using data of DDC treatment *vs.* control we built a quantitative model to explain changes of metabolites of the eicosanoid pathway due to treatment by DDC that mimics the NASH phenotype.

**Table 1:** Initial values of the arachidonic acid/eicosanoid metabolic model.

Symbol	AJ, control	AJ, DDC	B6, control	B6, DDC	PWD, control	PWD, DDC
PTGS1	1.00	2.13	1.00	1.41	1.00	2.09
ALOX5	1.00	1.00	1.00	1.00	1.00	1.00
PTGDS	1.00	1.00	1.00	1.00	1.00	1.00
STAT3	1.00	1.41	1.00	1.42	1.00	2.03
PKCD	1.00	4.30	1.00	4.48	1.00	3.85
GPX	1.00	6.51	1.00	11.70	1.00	19.42
PLA2	1.00	1.90	1.00	2.09	1.00	1.98
ALOX5AP	1.00	4.81	1.00	6.30	1.00	14.23
ALOX15	1.00	1.00	1.00	1.00	1.00	1.00
PC*	269.00	206.00	249.00	209.00	266.00	255.00

The enzyme concentrations of the control state were always initialized with 1.0 nM. The DDC initial value always reflects the fold change between the DDC-treated and the control expression value. The absolute concentration of phosphatidylcholine C33:2 (PC*) comes from MS-analysis and is measured in µM for control and DDC-treatment conditions of AJ, B6, and PWD mice.

### Model Optimization

Model parameters were optimized using experimentally determined metabolite concentrations of the model components. Metabolite concentrations were measured after 8 weeks of DDC treatment and interpreted as steady state concentrations for the model. The metabolites PGD2, 5-HPETE, 15-HPETE and 15-HETE have been found as being affected due to DDC treatment. These metabolites as well as upstream AA were used for model optimization. Therefore, the model was simulated into its steady state and the euclidean distance between simulated steady state concentrations and experimental metabolite data of the aforementioned metabolites were used in an objective function to optimize the kinetic parameters using COPASI. Parameter optimization was done using the metabolic concentrations of B6 control and B6 DDC mice. Kinetic parameters, i.e., the maximum reaction rates (V_max_), Michaelis-Menten constants (K_m_) and inhibition constants (K_i_) were optimized based on experimental data. The following five metabolites of the AA model were used to formulate an objective function: AA, 5-HPETE, and 15-hydroperoxyeicosatetraenoic acid (15-HPETE), 15-Hydroxyeicosatetraenoic (15-HETE), and prostaglandin D2 (PGD2). Let aforementioned metabolites be in a set M. The objective function *f* reads
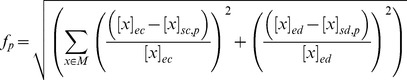
where, **p** is a set of kinetic parameters of the AA model. *ec* and *sc* represent experimental and simulated steady concentration in control condition, while *ed* and *sd* represent experimental and simulated steady concentration in the DDC-treated condition. The objective function *f* was minimized to obtain optimal kinetic parameters of the AA model using the genetic algorithm of COPASI with a population size of 50 and a generation size of 500. The identified kinetic parameters are listed in Table 2. The model is available in SBML format (suppl. File S1).

**Table 2:** Kinetic equations and their parameters of the arachidonic acid/eicosanoid metabolism model.

R1: PC → AA	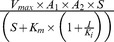	*V_max_* = 20.56 nM^2^s^−1^, *K_m_* = 2500 nM [BRENDA], *K_i_* = 100 nM, *A_1_* = {PLA2}, *A_2_* = {PERK}, *I* = [AA] nM, *S* = [PC] nM
R2: AA → 5-HPETE	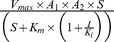	*V_max_* = 0.01 nM^2^s^−1^, *K_m_* = 0.0107 nM, *K_i_* = 8.603 nM, *A_1_* = {ALOX5}, *A_2_* = {ALOX5AP}, *I* = [5-HPETE] nM, *S* = [AA] nM
R3: 5-HPETE →	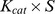	*S* = [5-HPETE], *K_cat_* = 0.0012s^−1^
R4: 5-HPETE → LTA4	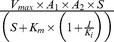	*V_max_* = 9.7953 nM^2^s^−1^, *K_m_* = 99.913 nM, *K_i_* = 0.709 nM, *A_1_* = {ALOX5}, *A_2_* = {ALOX5AP}, *I* = [LTA4] nM, *S* = [5-HPETE] nM
R5: LTA4 →	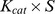	*S* = [LTA4] nM, *K_cat_* = 0.0012 s^−1^
R6: AA → 15-HPETE	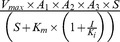	*V_max_* = 0.003 nM^3^s^−1^, *K_m_* = 0.067 nM, *K_i_* = 1.004 nM, *A_1_* = {ALOX15}, *I* = [15-HPETE] nM, *A_2_* = {PKCD}, *A_3_* = {PSTAT3}, *S* = [AA] nM
R7: 15-HPETE → 15-HETE	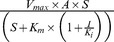	*V_max_* = 1.098 nMs^−1^, *K_m_* = 1.58 nM, *K_i_* = 0.0106 nM, *A_1_* = ALOX15, *I* = [15-HETE] nM, *S* = [15-HPETE] nM
R8:15-HETE →	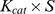	*S* = [15-HETE], *K_cat_* = 0.00127 s^−1^
R9: AA → PGH2	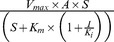	*V_max_* = 0.168 nMs^−1^, *K_m_* = 3.876 nM, *K_i_* = 0.013 nM, *A* = {PTGS1}*I* = [PGH2], *S* = [AA] nM
R10: PGH2 → PGD2	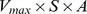	*V_max_* = 0.067 nMs^−1^, *A* = {PTGDS}, *S* = [PGH2]
R11:PGD2 →	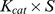	*S* = [PGD2] nM, *K_cat_* = 0.052 s^−1^
R12: AA →	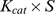	*S* = [AA] nM, *K_cat_* = 0.00096 s^−1^

*A*, *A_1_*, *A_2_* and *A_3_* are the ratios of gene expression or protein levels of the respective enzymes between DDC-treated vs. control mice. *S* is the substrate of the corresponding reaction. Squared brackets refer to concentration values and curly braces indicate fold changes of DDC treatment vs. control. The K_m_ value of PLA2 is taken from BRENDA [55], and other parameters of the table were fitted using the AJ mice metabolic concentrations (see results section).

**Figure 3:**
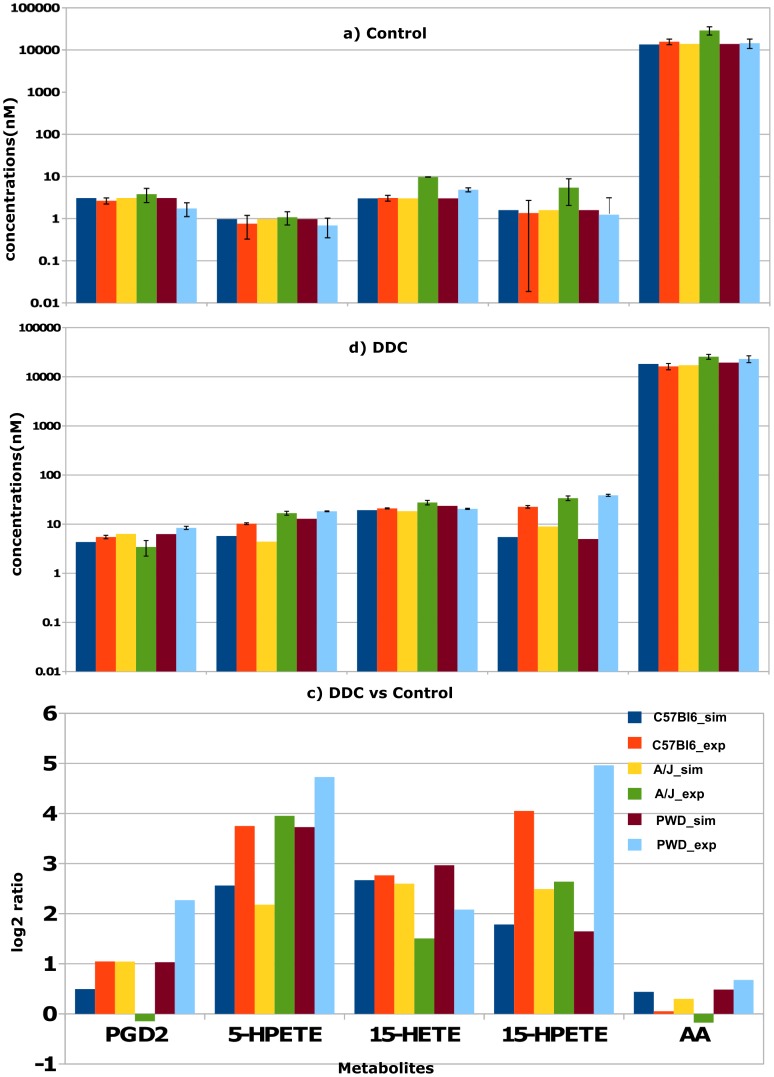
Comparison of simulated steady state and experimental metabolite concentrations. Quantitative data of simulated and experimental metabolite concentrations of all three mouse strains AJ, B6, and PWD for **a**) control **b**) DDC treatment, and **c**) their respective ratios of the metabolites prostaglandin D2, PGD2; 5- and 15-hydroperoxyeicosatetraenoic acid, 5- and 15-HPETE; 15-hydroxyeicosatetraenoic acid, 15-HETE; and arachidonic acid, AA.

Results of the fitted model for the metabolites AA, PGD2, 5-HPETE, 15-HPETE, and 15-HETE using the trained B6 model were compared with the experimental results (Fig. 3; C57Bl6_sim vs. C57Bl6_exp). Except of AA all of the aforementioned metabolites were up-regulated (>1.5 fold) in both, experimental and simulated data, due to DDC in B6 mice (Fig. 3c). AA was not found altered in the experimental data set, but was slightly up-regulated in the simulation (1.35-fold).

For validation of the model we predicted DDC-induced metabolite changes for AJ and PWD using the trained model. Simulation results predict an up-regulation (>1.5-fold) of 5-HPETE, 15-HPETE and 15-HETE in both strains, which was in line with the experimental data. The concentration of PGD2 was found increased (>1.5-fold) between experimental and simulated data for PWD (Fig. 3c). In AJ our modeling approach predicts a 2-fold increase of PGD2 concentration in response to the DDC-treatment, but the experimental data showed no changes (0.9 fold). This disagreement can be strain-specific because one can expect changes in the concentration of PGD2 due to an up-regulated gene expression of *Ptgs1* which is located upstream in the metabolic pathway. AA was up-regulated (> = 1.5-fold) in DDC-PWD mice, which is concordant with the experimental data, while for AJ AA is not found as being changed (0.9 fold) in the experimental data, whereas a minor up-regulation (1.2-fold) was predicted by the simulation (Fig. 3c).

### Key regulatory enzymes of the DDC mouse model

**Figure 4:**
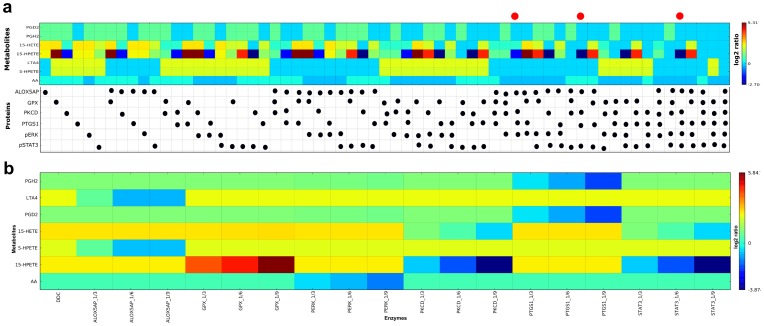
Identification of key regulatory enzymes (a) and drug testing (b) of the arachidonic acid/eicosanoid metabolism model. **a**) Key enzymes or enzyme combinations of the DDC model condition of AJ were reverted to control conditions of this strain to judge the effect on the change of the metabolite state. Black dots indicate enzymes or enzyme combinations that were reverted to control conditions. Red dots indicate those enzyme combination that are able to bring back the DDC-treated state to control conditions. **b**) *In silico* drug testing of the model by simulating down-regulation of individual enzyme concentrations as given by their respective expression value by 1/3rd-, 1/6th- and 1/9th of the DDC-treated state of AJ.

To identify key regulatory enzymes of the AA/eicosanoid pathway in DDC treated mice, a kind of sensitivity analysis of the trained model was performed. Starting with DDC-treated mice of the AJ strain, each enzyme or enzyme combinations were reverted to its/their activity in normal, untreated condition. This analysis was performed for ALOX5AP, GPX, PKCD, PTGS1, pERK, and pSTAT3 whose activity was found to be perturbed due to DDC-treatment. A reference state was defined where the activity of all enzymes is equal to the normal condition. Results of this analysis are depicted in Fig. 4a. Reverting a single enzyme or combinations of two enzymes were not sufficient to bring the DDC-treated state back to normal because it affects only some branches of the model, as for example a change in the activity of ALOX5 has an effect on the regulation of the downstream metabolites 5-HPETE and LTA4 (cf. Fig. 2). We found that the combinations of the enzymes ALOX5AP, PKCD and PTGS1 with either pERK or pSTAT3 can bring the DDC-treated metabolic state back to normal, with the exception of 15-HETE and AA. Only the combination ALOX5AP, PERK, PKCD, STAT3, and PTGS1 or the combination of all six enzymes was able to bring back the DDC-treated state to normal.

### Simulating drug effects in the DDC mouse model

To study potential drug targets for steatosis or inflammation *in silico* drug tests were performed. For instance, cyclooxygenase-2 (PTGS2/COX-2) is a frequent target of anti-inflammatory drugs [42]. In the computer simulation, the activity of each enzyme of the AJ DDC model was inhibited by 3-, 6- and 9-fold, respectively. The calculated effects of those enzyme inhibitions on the regulation of metabolites are shown in Fig. 4b. The inhibition of PTGS1 and ALOX5AP leads to a down-regulation of the respective downstream components (either PGH2 and PGD2 or 5-HPETE and LTA4). ALOX15 is activated by PKCD and pSTAT3, and the inhibition of PKCD or pSTAT3 leads to a down-regulation of the downstream components 15-HPETE and 15-HETE. It can be expected that the inhibition of glutathione peroxidase (GPX) leads to a down-regulation of the downstream component 15-HETE, but the model predicts an up-regulation of 15-HPETE and no change in 15-HETE. This is due to the complex regulation of ALOX15, i.e., an activation of the complex PKCD and pSTAT3 and an inhibition of GPX by 15-HETE.

## Discussion

Using a DDC-based mouse model of steatohepatitis we analyzed three different mouse strains AJ, B6, and PWD covering a broad range of genetic variations. AJ, B6, and PWD mice respond with different degrees of steatohepatitis ranging from high, low to resistant, respectively. Based on gene expression and metabolic data we identified differences in hepatic SAMe metabolism in respect to the different steatohepatitis phenotypes across the individual mouse strains. SAMe metabolism might explain susceptibility of AJ mice. SAMe is a key methyl-group donor for phosphatidylcholine synthesis that is required, e.g., for the export of very-low-density lipoproteins (VLDL) and triglycerides from the liver [43]. Furthermore, VLDL synthesis has been found to be impaired by MAT1A-knockout that could be recovered by SAMe administration in MAT1A deficient mice [43]. SAMe and methionine metabolism was found to be perturbed in NASH patients [44] and it may play an important role in development of NAFLD, such as NASH [30]. A study suggests depletion of hepatic anti-oxidants (e.g. reduced glutathione and SAMe) promotes oxidative stress and may induce cellular alterations typical for steatohepatitis [6].

We identified AA metabolism as being the most affected one upon all of the three mouse strains and we propose a dynamic model of AA metabolism of NASH-like phenotypes in mouse induced by DDC. The fitted model provides good predictions between the experimental and simulated metabolic data using the respective gene expression data.

To determine regulatory roles of important enzymes in the upregulation of metabolic levels of the AA/eicosanoid pathway due to DDC-treatment, a sensitivity analysis was performed by reverting to the activity of the enzymes to normal, untreated conditions. The simulated combination of the enzymes ALOX5AP, PERK, PKCD, STAT3, and PTGS1 is able to revert the DDC-treated metabolic state to normal. This multi-targets inhibition might likely become a future approach in respect to individualized medicine as it has already been suggested for cancer [45].

Furthermore, we did *in silico* drug testing of several model enzymes. Inhibition of PTGS1 and ALOX5AP caused down-regulation of PGH2 and PGD2 or 5-HPETE and LTA4, respectively. These enzymes are known targets to treat inflammation. PTGS1 (COX-1), for instance, can be inhibited by mofezolac, SC-560, and other drugs [46]. Inhibition of ALOX5AP also known as 5-lipoxygenase activating protein, or FLAP may be useful in the prevention of hepatotoxin-induced necro-inflammatory injury [47]. Drug molecules can interact with multiple targets to alter the state and function of the associated biological network. Licofelone is a novel 5-LOX/COX-inhibitor which inhibits two enzymes to avoid side effects [48]. Overall, the dynamics of the AA model can be used for *in silico* drug studies to test multiple drugs and potential drug targets. The model confirmed the upregulation of PGD2 due to DDC treatment found experimentally. This could be linked to the key transcription factors/ligand-activated nuclear receptors such as PPARδ which has been implicated as a key regulator of energy homeostasis and may represent future research avenues to study the interaction of metabolic and signaling pathways [49].

Prostaglandin E2 (PGE2) was found to be upregulated in AJ and B6 mice but not in PWD (Fig. 1f). PGE2 promotes inflammation after binding to prostaglandin E receptor 2 (EP2) [50] and an inflammatory marker tumor necrosis factor alpha (TNFα) regulates NASH development in a diet-induced mouse model [51]. To explain inflammation phenotypes through signaling by PGE2-EP2-TNFα we observed an upregulation of *Tnf* expression (35-fold) in AJ and B6 mice. Upregulation of PGE2 and TNFα in AJ and B6 mice may explain inflammation of the NASH phenotype.

The spectrum of NAFLD can be characterized by specific alterations in hepatic lipid composition. A comprehensive analysis of plasma lipids and eicosanoids in human revealed a stepwise increase in lipoxygenease metabolites 5-HETE, 8-HETE and 15-HETE in NAFLD [52]. This correlates with our observations in the experimental and simulated data, where concentrations of 5-HETE and 15-HETE were increased in DDC mice. Another study reported overexpression of cyclooxygenase-2 (COX-2) in hepatocellular carcinoma (HCC) patients [53]. Using immunohistochemistry they studied COX-2 overexpression in different chronic liver diseases including NASH, chronic hepatitis, and liver cirrhosis. In our study, we detected overexpression of PTGS1 (COX-2) in DDC mice, which is a key regulator of prostaglandin formation.

Martínez-Clemente et al. demonstrated that hyperlipidemia-prone apolipoprotein E-deficient (ApoE(−/−)) mice exhibit hepatic steatosis and increased susceptibility to hepatic inflammation and advanced fibrosis [54]. They found in an experimental model the proinflammatory 5-lipoxygenase (5-LO) pathway to be up-regulated and thus causing liver inflammation and fibrogenesis. They also found that the inhibition of the 5-LO pathway results in a significant reduction in liver inflammation. Our data supports an up-regulation of ALOX5AP through 5-LO pathway due to DDC treatment in AJ, B6, and PWD, leading to an upregulation of the downstream component 5-HPETE in the model that is supported by our experimental data.

In conclusion, mRNA expression data combined with mathematical modeling of metabolic systems provides a useful tool to better understand cellular metabolism although the correlation between transcripts and proteins can deviate depending on cellular location, biological function, and organism [13]. The development of NAFLD is characterized by broad changes on the molecular level. Detailed analysis of three differently susceptible mouse strains, which reflect genetic diversity in humans, showed major deregulation of arachidonic acid metabolism. Detailed modeling of the arachidonic acid metabolism and model predictions of metabolic levels are in good agreement with experimental profiles when the model is initialized by the measured gene and protein expression data. The study identified deregulated genes, proteins, metabolites and affected pathways of NAFLD etiology in mouse and serves as an integrated resource of omics data for the development of computational models of the disease, such as AA and SAMe metabolism.

## Supporting Information

Table S1 **Susceptible genes (first column: only differentially expressed in AJ) and all differentially expressed genes of AJ, B6 and PWD mice due to DDC treatment.**
(XLS)

Table S2 **RNA-Seq expression data (RPKM values) of all three mice that were used for the principle component analysis (PCA analysis).**
(XLS)

Table S3 **Differentially expressed genes irrespective of strain.**
(XLS)

Table S4 **Results from RPPA analysis of a set of selected proteins.**
(XLS)

Table S5 **Metabolic profiles of a set of metabolites and t-test results for identification of metabolic differences.**
(XLS)

Table S6 **Results of pathway over-representation analysis of susceptibility genes using ConsensusPathDB.**
(XLS)

Table S7 **288 differentially expressed metabolic genes of the genome-scale metabolic network that were identified by edgeR over all three strains that were subsequently used in pathway over-representation analysis.**
(XLS)

Table S8 **Pathway over-representation analysis based on suppl. Table S7 using ConsensusPathDB.**
(XLS)

Table S9 **Pathway over-representation analysis based on metabolic profiles using ConsensusPathDB.**
(XLS)

Table S10 **Identification of differentially expressed genes between control and DDC-treatment over all strains of all genes of the AA model.**
(XLS)

File S1 **SBML file of the arachidonic acid/eicosanoid metabolic model.**
(SBML)
